# Cytogenetic and molecular genotyping in the allotetraploid *Festuca pratensis* × *Lolium perenne* hybrids

**DOI:** 10.1186/s12864-019-5766-2

**Published:** 2019-05-14

**Authors:** Joanna Majka, Katarzyna Bzdęga, Agnieszka Janiak, Hanna Ćwiek-Kupczyńska, Paweł Krajewski, Tomasz Książczyk, Zbigniew Zwierzykowski

**Affiliations:** 1 Department of Environmental Stress Biology, Institute of Plant Genetics, Polish Academy of Sciences, Poznań, Poland; 2 Department of Botany and Nature Protection, University of Silesia in Katowice, Katowice, Poland; 3 Department of Genetics, University of Silesia in Katowice, Katowice, Poland; 4 Department of Biometry and Bioinformatics, Institute of Plant Genetics, Polish Academy of Sciences, Poznań, Poland

**Keywords:** *Festuca*, *Festulolium* hybrids, *Lolium*, Genomic composition, Inheritance pattern, Rearrangements, Recombination

## Abstract

**Background:**

Species of the *Festuca* and *Lolium* genera, as well as intergeneric *Festuca* × *Lolium* (*Festulolium*) hybrids, are valuable fodder and turf grasses for agricultural and amenity purposes worldwide. *Festulolium* hybrids can merge in their genomes agronomically important characteristics. However, in polyploid plants, especially in allopolyploids, the hybridization of divergent genomes could contribute to various abnormalities, such as variability in chromosome number, structural rearrangements, and/or disorders in inheritance patterns. Here we studied these issues in allotetraploid *Festuca pratensis* × *Lolium perenne* hybrids.

**Results:**

Cytogenetic procedures, including fluorescent in situ hybridization, genomic in situ hybridization, and molecular markers – inter-simple sequence repeats (ISSR) were exploited. This cytogenetic approach indicated the dynamics in the number and distribution of ribosomal RNA genes and structural rearrangements for both parental genomes (*Festuca* and *Lolium*) in hybrid karyotypes. The separate analysis of *F. pratensis* and *L. perenne* chromosomes in hybrid plants (F_2_-F_3_ generations of *F. pratensis* × *L. perenne*) revealed the asymmetrical level of rearrangements. Recognized structural changes were mainly located in the distal part of chromosome arms, and in chromosomes bearing ribosomal DNA, they were more frequently mapped in arms without this sequence. Based on the ISSR markers distribution, we found that the tetrasomic type of inheritance was characteristic for the majority of ISSR loci, but the disomic type was also observed. Nonetheless, no preference in the transmission of either *Festuca* or *Lolium* alleles to the following generations of allotetraploid *F. pratensis* × *L. perenne* hybrid was observed.

**Conclusion:**

Our study reports cytogenetic and molecular genotyping of the *F. pratensis* × *L. perenne* hybrid and its following F_2_-F_3_ progenies. The analysis of 137 allotetraploid *F. pratensis* × *L. perenne* hybrids revealed the higher level of recombination in chromosomes derived from *F. pratensis* genome. The results of ISSR markers indicated a mixed model of inheritance, which may be characteristic for these hybrids.

**Electronic supplementary material:**

The online version of this article (10.1186/s12864-019-5766-2) contains supplementary material, which is available to authorized users.

## Background

Polyploids frequently occur within the plant kingdom, especially in domesticated and economically important plants in the world [1]. Within polyploids, two basic types auto- and allopolyploids are usually. Genomes of allopolyploids contain chromosome sets from two or more divergent genomes of donor plants. In the course of evolution, plant genomes underwent various processes that had an impact on numerical and structural changes [2, 3]. The comparison of karyotypes within the grass family and various genera revealed vast variability in the chromosome number and in their morphology. There are three different levels of changes that may occur in the hybrid genomes: (*i*) DNA sequence (e.g., allelic differences caused by nucleotide substitutions or indels), (*ii*) structure and gene order (e.g., chromosomal rearrangements), and (*iii*) total number of chromosomes in the complement. In allopolyploids, especially in the newly formed, smaller (at DNA sequence level) and larger (at chromosome level) genetic changes could result from recombination between homoeologous chromosomes. Pairing affinity and chiasmata formation between homoeologous chromosome sets have an enormous impact on inheritance patterns [4–6]. Importantly, several gametophytic and zygotic barriers have been reported, which caused deviation of allele frequencies from Mendelian ratios, for example in the interspecific crosses in rice [7].

Variation in a number of chromosomes and/or in their structure are pivotal issues in the crossing of plants derived from different genera. Plants with the expected chromosome number, as well as aneuploids may be observed within the hybrid’s progeny [8–10]. Recombinant chromosomes can be characterized using molecular and cytogenetic tools, such as genetic markers (e.g. [11, 12]) and genomic in situ hybridization (GISH) technique (e.g. [9, 13–17]). The application of GISH, usually combined with fluorescent in situ hybridization (FISH), enables the characterization of the genome composition of hybrid plants providing valuable information for breeding new cultivars on the early stages of material selection.

*Lolium* species (*L. perenne* and *L. multiflorum*), the major source of forage in temperate regions of the world, depict the considerable variation of quality traits, crucial implication for grass breeding programs. This group of plants is characterized by high digestibility, palatability and intensive spring growth [18]. Traits which are of great importance for adaptation to abiotic as well as biotic stresses, e.g. drought tolerance, winter hardiness and resistance to fungal diseases, are provided by *Festuca* species (*F. pratensis* and *F. arundinacea*) [18]. Desirable traits from *Lolium* species can be merged with features of *Festuca* species through crosses and production of intergeneric *Festuca* × *Lolium* hybrids (*Festulolium*). Chromosomes of these hybrids can recombine at high frequency [9, 11, 19].

The main aims of this study were the examination of the genomic composition of *F. pratensis* × *L. perenne* hybrid and its following generations (F_2_-F_3_) and the determination of inheritance pattern in this plant material. To achieve these goals, we applied the cytogenetic procedures, including FISH with ribosomal DNA (rDNA) sequences to recognize selected chromosomes in the complements, GISH to analyze the genomic architecture, and molecular markers – inter-simple sequence repeats (ISSR) to determine the types of ISSR loci inheritance in three successive generations of *F. pratensis* × *L. perenne*.

## Results

### Karyotype analysis of *F. pratensis* × *L. perenne* hybrids

**Fig. 1:**
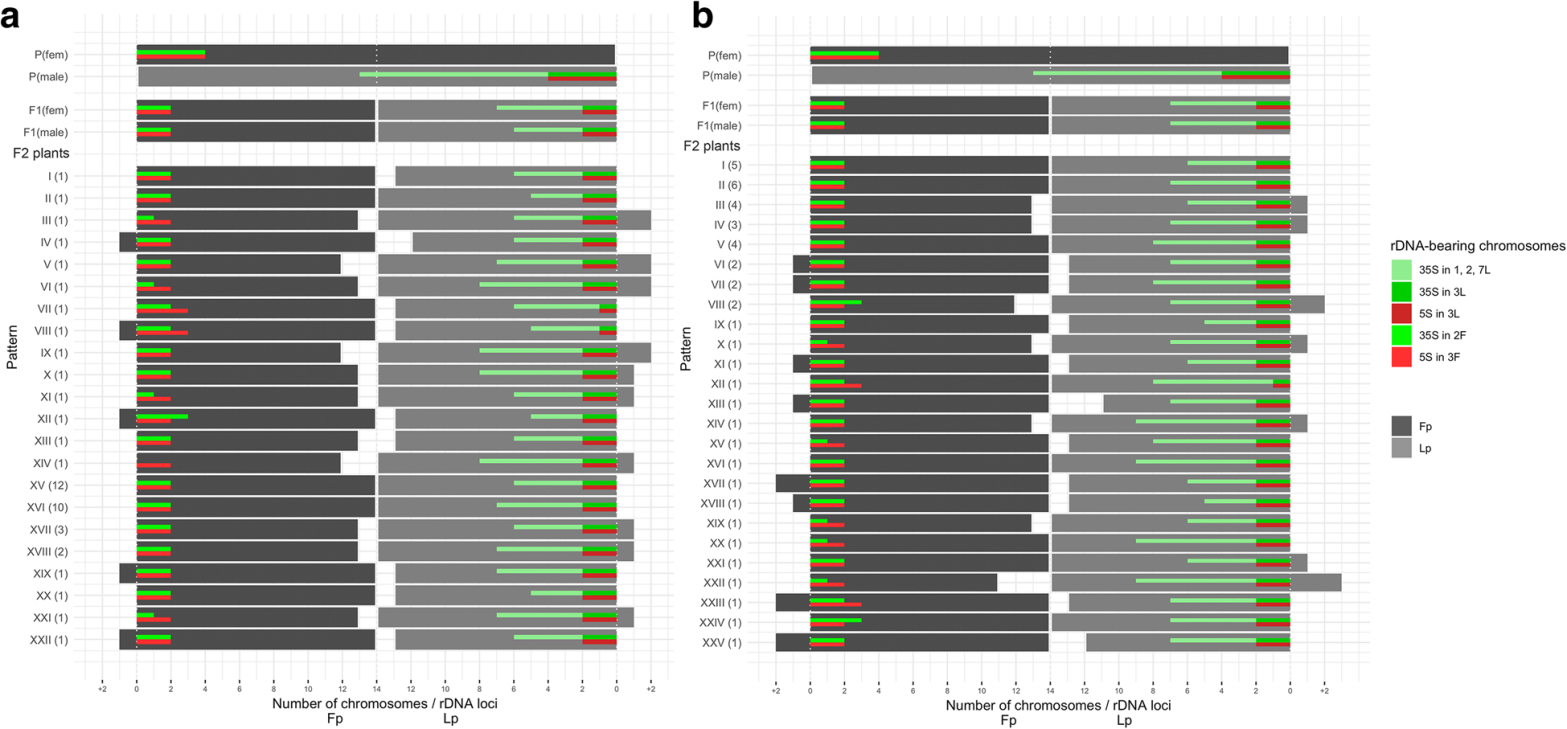
The distribution of the total number of chromosomes in karyotypes and rDNA-bearing chromosomes in 90 plants of F_2_ generation of *F. pratensis* × *L. perenne* hybrid – in combination no. 1 (**a**) and in combination no. 2 (**b**). At the top of both graphs were shown results for parents – two parental plants: P (Fp 11/59) (♀), P (Lp 08/33) (♂); four F_1_ plants: two plants – F_1_ 6-2A (♀), F_1_ 6-7A (♂) for combination no. 1 of F_2_, and two plants – F_1_ 6-2A (♀), F_1_ 6-3B (♂) for combination no. 2 of F_2_. On the *y*-axis obtained rDNA patterns (Roman numerals) and their frequency in the brackets (Arabic numerals) were shown. On *x*-axis were presented: a total number of chromosomes divided into two groups – *Festuca* and *Lolium* chromosomes, as well as the number of particular rDNA-bearing chromosomes

**Fig. 2:**
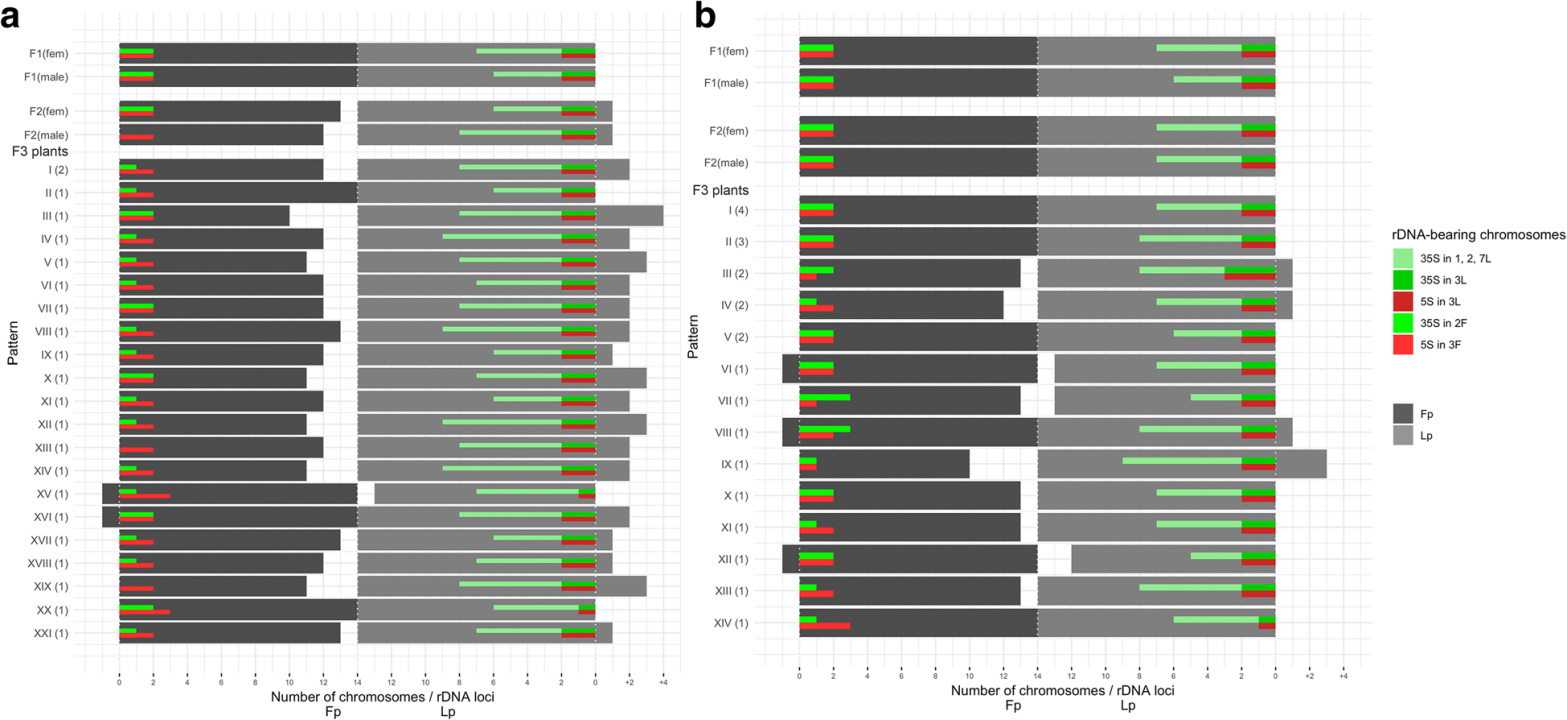
The distribution of the total number of chromosomes in karyotypes and rDNA-bearing chromosomes in 44 plants of F_3_ generation of *F. pratensis* × *L. perenne* hybrid – in combination no. 3 (**a**), and in combination no. 4 (**b**). At the top of both graphs were shown results for parents – two F_1_ plants: F_1_ 6-2A (♀), F_1_ 6-7A (♂) and four plants of F_2_: two plants – F_2_ 2A-15 (♀), F_2_ 2A-5 (♂) for combination no. 3 of F_3_, and two plants – F_2_ 2A-27 (♀), F_2_ 2A-12 (♂) for combination no. 4 of F_3_. On the *y*-axis obtained rDNA patterns (Roman numerals) and their frequency in the brackets (Arabic numerals) were shown. On *x*-axis were presented: a total number of chromosomes, divided into two groups - *Festuca* and *Lolium* chromosomes, as well as the number of particular rDNA-bearing chromosomes

In total, 134 plants of F_2_ (combinations no. 1 and 2) and F_3_ (combinations no. 3 and 4) generations were analyzed (see Additional file 1: Figure S1) and the majority of them were tetraploid with 2n = 4x = 28 (83.3% in F_2_ and 68.2% in F_3_). Among aneuploid plants, both hypoploids (2n = 26, 27) and hyperploids (2n = 29, 30, 31) were recorded. Hypoploids constituted 11.1% in F_2_ and 25% in F_3_, while hyperploids – 5.6% and 6.8%, respectively (Figs. 1, 2).

Allotetraploid plants with 14 chromosomes of *F. pratensis* and 14 chromosomes of *L. perenne* were distinguished in 56% of F_2_ and 40% of F_3_ generation. Within the remaining plants with 28 chromosomes, the genotypes with the higher number of *L. perenne* chromosomes were predominated (29.3% in F_2_ and 53.3% in F_3_). Plants with lower (2n < 28) and higher (2n > 28) total number of chromosomes, that constituted 16.7% in F_2_ and 31.8% in F_3_, were also characterized by the higher number of *L. perenne* chromosomes (average 43.3% for both generations) (see Additional file 2: Table S1).

### rDNA-FISH mapping

rDNA-FISH mapping revealed that *F. pratensis* and *L. perenne* species, used to obtain F_1_ hybrids, had four loci of 5S rDNA. In the case of 35S rDNA, four and 15 loci were recorded, respectively. Among F_1_ plants, 5S rDNA sequence was mapped to four chromosomes in the genomes of maternal plant and both pollinators. Whereas 35S rDNA was hybridized to eight sites in the karyotype of pollinator 6-7A and nine sites in the female plant and pollinator 6-3B (Fig. 1). Four F_2_ plants used to produce two combinations of F_3_ (no. 3 and 4) were characterized by four loci of 5S rDNA and eight loci of 35S rDNA (combination no. 3), as well as four loci of 5S rDNA and nine loci of 35S rDNA (combination no. 4) (Fig. 2). Among F_2_ progeny, the number of 5S rDNA loci was equal to four, except for one plant with five loci of this sequence (Fig. 1b). This sequence was located in an interstitial part of chromosome 3F and near the centromeric region of chromosome 3 L. In the vast majority of F_2_ individuals, there were two pairs of each chromosome types, in minority three chromosomes 3F and only one chromosome 3L (see Additional file 2: Table S1). The similar trend was observed among F_3_ plants – the number of 5S rDNA equaled four, with the exception of one plant with three loci of 5S rDNA (Fig. 2b). In all F_3_ analyzed hybrids, two chromosomes 3F and two 3L were mostly observed. Only for several plants, three 3F and one 3L chromosomes as well as conversely one 3F and three 3L chromosomes were recorded (Fig. 2). In the karyotype of plants with three loci of 5S rDNA, one chromosome 3F and two chromosomes 3L were observed, while for the karyotype with five loci of this sequence three 3F and two 3L chromosomes were noticed.

The number of 35S rDNA sequence was more variable and ranged from seven to eleven loci in F_2_ and F_3_ plants (Figs. 1, 2). This sequence was located at the secondary constriction regions of 2F and 3L chromosomes, as well as on the chromosomes 1, 2 and 7L. In both generations, the variation in the number of particular chromosomes was as follows: 3-7 signals for chromosomes 1, 2, 7L, 1-3 for chromosomes 3L, and 0-3 for chromosomes 2F (Figs. 1, 2).

With regard to the type and the number of recognized rDNA-bearing chromosome pairs, the analysis showed 22 and 25 various rDNA patterns in combination no. 1 and 2 of F_2_ generation, respectively (Fig. 1). Whereas in F_3_ generation for combinations no. 3 and no. 4, subsequently 21 and 14 rDNA patterns were detected (Fig. 2). The most frequent patterns (48.9%) in F_2_ combination no. 1 reflected rDNA arrangements occurring in parental plants. In the remaining F_2_ and F_3_ plants, different rDNA profiles were identified. However, the most repeated patterns were connected with one more or one less 35S rDNA locus. The analysis of the total number of rDNA-bearing chromosomes for all F_2_ and F_3_ analyzed plants revealed that even number of chromosomes prevailed, what was expected, however, with the exception of the number of chromosomes 2F. In F_3_ combination no. 3, almost 70% of plants had in karyotype only one chromosome 2F (Fig. 2).

### GISH mapping

To reveal rearrangements in the karyotypes of *F. pratensis* × *L. perenne* hybrids, the analysis with total genomic DNA (GISH) was performed and this approach did not reveal any structural changes in F_1_ plants. The F_2_ and F_3_ progenies of the *F. pratensis* × *L. perenne* hybrid were characterized by karyotype rearrangements. Their number increased from generation to generation and was as follows: 179 for F_2_ plants and 217 for F_3_ plants (see Additional file 2: Table S1). These numbers include the terminal, interstitial and pericentromeric types of rearrangements in total. The rearranged chromosomes were divided into two groups consisting of rearranged rDNA-bearing chromosomes and rearranged non-rDNA-bearing ones. The level of rearrangements in rDNA-bearing chromosomes equaled 38.8% in F_2_ and 47% in F_3_ generation, while in non-rDNA-bearing chromosomes 61.2% and 53%, respectively. Among identified rearrangements, terminal (92.2%), interstitial (7.1%) and pericentromeric (0.7%) types were recognized. Non-rDNA-bearing chromosomes were more frequent (61.4% in F_2_ and 63.2% in F_3_) in the group of non-recombined chromosomes (see Additional file 2: Table S1).

**Fig. 3:**
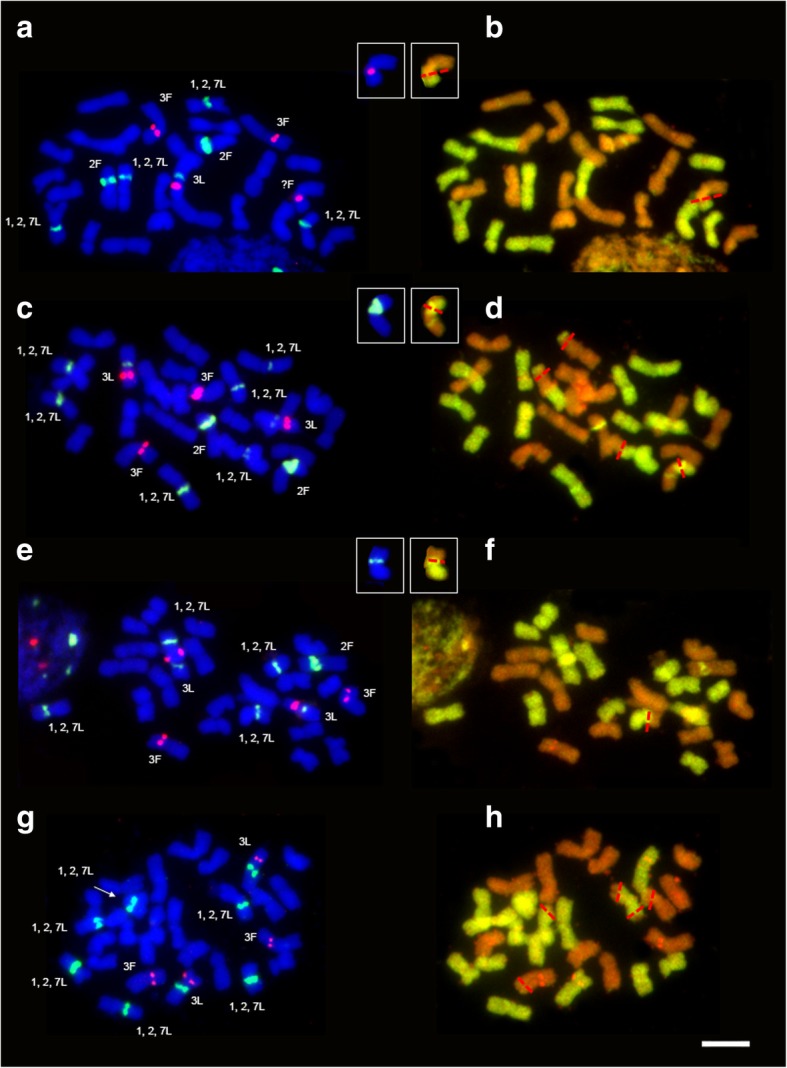
Rearrangements in rDNA-bearing chromosomes among *F. pratensis* × *L. perenne* hybrids. (**a**, **b**) F_2_ 2A-33 with recombined 3L/non-rDNA-bearing *Festuca* chromosome; (**c**, **d**) F_2_ 2B-21 with recombined 2F chromosome; (**e**, **f**) F_2_ 2B-28 with recombined one of 1, 2, 7L group of chromosomes, and (**g**, **h**) F_2_ 2A-5 without 2F chromosomes. (**a**, **c**, **e**, **g**) FISH with 5S rDNA (red) and 35S rDNA (green); chromosomes were counterstained with DAPI (blue). (**b**, **d**, **f**, **h**) GISH with total genomic DNA of *L. perenne* as a probe (green) and with blocking genomic DNA of *F. pratensis* (orange) on the same metaphase plate; chromosomes were counterstained with propidium iodide (orange). Red dotted lines indicate the place of rearrangement. Chromosomes with rearrangements corresponding with rDNA position were exemplified in the insets. Scale bar 5 μm

The separate analysis of rearranged chromosomes derived from *F. pratensis* and *L. perenne* genomes revealed that the number of recombined rDNA-bearing chromosomes was on a similar level for F_2_ plants, 51.7% and 48.3% for *F. pratensis* and *L. perenne* genome, respectively. More differences were found in F_3_ generation, 40.4% for *F. pratensis* and 59.6% for *L. perenne*. When both generations were analyzed together, slightly predominance of the rearranged rDNA-bearing chromosomes of *L. perenne* (55.5%) was observed. On the other hand, in non-rDNA-bearing chromosomes, the higher level of recombination was detected for *F. pratensis* chromosomes (67.2% in F_2_ and 76.7% in F_3_). Additionally, among analyzed plants with detected structural rearrangements, several genotypes with the uncommon location of changes were noticed. In these plants, the rearrangements colocalized with the position of rDNA locus (5S and 35S rDNA) in various type of rDNA-bearing chromosomes – chromosome 3L/non-rDNA-bearing *Festuca* chromosome (Fig. 3a and b), chromosome 2F (Fig. 3c and d), and chromosomes 1, 2, 7L (Fig. 3e and f). The similar position of rearrangement was also detected in chromosome 3F. Furthermore, an F_2_ plant without two 2F chromosomes was observed (Fig. 3g and h).

**Fig. 4:**
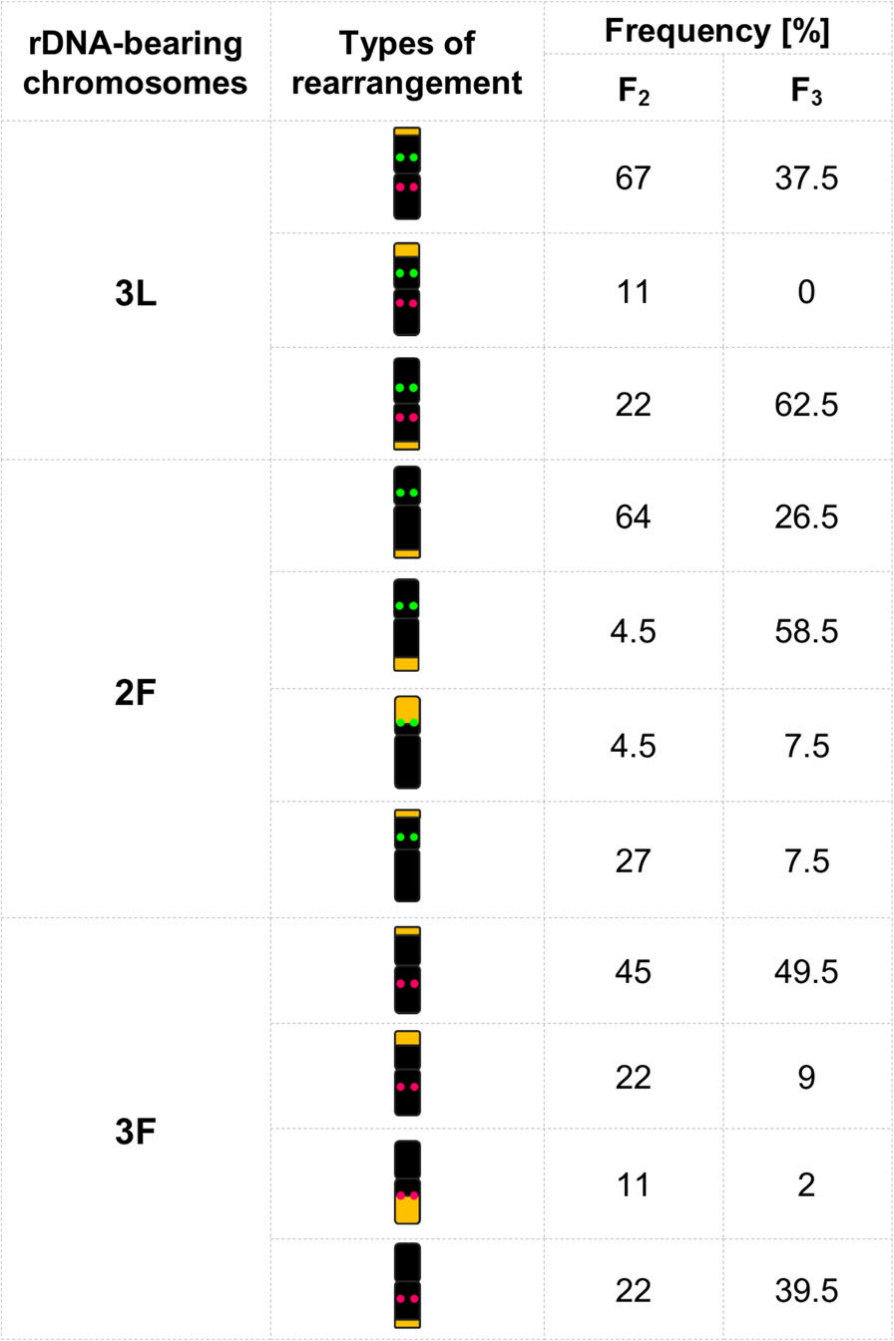
The distribution and the frequency of rearrangements in rDNA-bearing chromosomes (3L, 2F, and 3F) among F_2_ and F_3_ generations of *F. pratensis* × *L. perenne* hybrid*.* Green dots on the chromosomes illustrate 35S rDNA loci; pink dots illustrate 5S rDNA; orange blocks illustrate the position and size of rearrangements

The detailed analysis of recombined rDNA-bearing chromosomes in F_2_ and F_3_ plants unveiled the types and the frequency of observed changes. Rearranged regions were mostly located in the arms without rDNA-marker for both parental genomes and both generations and they always were located in the distal parts of chromosomes (Fig. 4). In F_2_ plants, the most recombined types were: chromosomes 2F and the group of chromosomes 1, 2, 7L, while in F_3_ – the group of chromosomes 1, 2, 7L and chromosomes 3F (see Additional file 2: Table S1). However, the types of rearrangements and their frequencies in the group of chromosomes 1, 2, 7L were not shown in Fig. 4, because the location of 35S rDNA and similar size of chromosomes do not allow to distinguish them, therefore we could not determine precisely the type and the frequency of changes in each pair of chromosomes separately. In the F_2_ generation, the highest level of recombination was recorded for 35S rDNA-bearing arm of chromosomes 3L. In the F_3_ generation, however, more rearrangements were found in the opposite location – in arm bearing 5S rDNA sequence. For chromosomes 2F and 3F, the majority of changes in the arms without rDNA landmarks were occurred (Fig. 4).

### Statistical analysis of cytogenetic traits

Statistical analysis of selected cytogenetic traits in F_2_ generation revealed weakly significant differences between distributions and strongly significant differences between mean values for the number of 35S rDNA in *L. perenne* genome what is related to the number of chromosomes 1, 2, 7L (Table 1). The analysis of F_2_ plants and the comparison between F_2_ and F_3_ generations revealed the statistically significant differences for the number of non-rDNA-bearing chromosomes (Tables 1 and 2). In comparison between generations, it was also revealed that the statistically significant difference existed for the number of 35S rDNA in *F. pratensis* genome what is related to the number of chromosomes 2F (Table 2).

**Table 1:** Comparison of nine selected cytogenetic trait distributions in F_2_ generation of *F. pratensis* × *L. perenne* hybrid

Trait	Value of the trait for F_1_ parental plants			*P*-value for equality of distributions in F_2_ generation^a^	Mean value in F_2_ generation		*P*-value for equality of mean values in F_2_ generation^b^	*P*-value for mean within generation being equal to mid-parent value^c^	
	F_1_ 6-2A	F_1_ 6-7A	F_1_ 6-3B		Combination no. 1	Combination no. 2		Combination no. 1	Combination no. 2
Number of 35S rDNA in Lp genome	7	6	7	**0.057**	6.42	6.98	**0.005**	0.509	0.884
Number of 35S rDNA in Fp genome	2	2	2	0.656	1.89	1.96	0.465	0.096	0.486
Number of 5S rDNA in Lp genome	2	2	2	1.000	1.96	1.98	0.562	0.160	0.323
Number of 5S rDNA in Fp genome	2	2	2	1.000	2.04	2.04	1.000	0.160	0.160
Number of 1, 2, 7L chromosomes	5	4	5	**0.075**	4.47	5.00	**0.007**	0.769	1.000
Number of 3L chromosomes	2	2	2	1.000	1.96	1.98	0.562	0.160	0.323
Number of 2F chromosomes	2	2	2	0.675	1.89	1.96	0.465	0.096	0.486
Number of 3F chromosomes	2	2	2	1.000	2.04	2.04	1.000	0.160	0.160
Number of chromosomes M-	17	18	16	**0.024**	17.53	17.00	**0.009**	0.785	**0.003**

(Lp) *L. perenne*; (Fp) *F. pratensis*; (M+) rDNA-bearing chromosome; (M-) non-rDNA-bearing chromosome; (m+) chromosome arm with rDNA sequence; (m-) chromosome arm without rDNA sequence; bold *P*-values for comparisons considered as statistically significant

^a^*P*-value for the χ^2^ test of equal distributions of the trait in F_2_ combinations no.1 and 2 (based on permutations)

^b^*P*-value for the t-test of equality of mean values for the trait in F_2_ combinations no. 1 and 2

^c^*P*-value for the t-test of equality of mean value in F_2_ generation and mean of parents

**Table 2:** Comparison of nine selected cytogenetic trait distributions in F_2_ and F_3_ generations of *F. pratensis* × *L. perenne* hybrid

Trait	Value of the trait for F_2_ parental plants				*P*-value for equality of distributions in F_2_ and F_3_ plants	Mean value in generation		P-value for equality of mean values in F_2_ and F_3_ plants
	2A-15	2A-5	2A-27	2A-12		F_2_	F_3_	
Number of 35S rDNA in Lp genome	6	8	7	7	**0.002**	6.42	7.32	**< 0.001**
Number of 35S rDNA in Fp genome	2	0	2	2	**< 0.001**	1.89	1.46	**< 0.001**
Number of 5S rDNA in Lp genome	2	2	2	2	0.239	1.96	1.98	0.718
Number of 5S rDNA in Fp genome	2	2	2	2	0.123	2.04	1.98	0.328
Number of 1, 2, 7L chromosomes	4	6	5	5	**< 0.001**	4.47	5.32	**< 0.001**
Number of 3L chromosomes	2	2	2	2	0.245	1.96	1.98	0.718
Number of 2F chromosomes	2	0	2	2	**< 0.001**	1.89	1.48	**< 0.001**
Number of 3F chromosomes	2	2	2	2	0.105	2.04	1.98	0.328
Number of chromosomes M-	18	17	17	17	**0.040**	17.53	17.11	**0.027**

(Lp) *L. perenne*; (Fp) *F. pratensis*; (M+) rDNA-bearing chromosome; (M-) non-rDNA-bearing chromosome; (m+) chromosome arm with rDNA sequence; (m-) chromosome arm without rDNA sequence; bold *P*-values for comparisons considered as statistically significant

We also studied separately the chromosomes derived from *F. pratensis* and *L. perenne* genomes, divided into groups: rearranged and non-rearranged, as well as rDNA-bearing and non-rDNA-bearing chromosomes. Rearranged chromosomes with rDNA sequence, were subdivided into two classes based on the location of rearrangements – in the arm with rDNA and without rDNA landmark (Table 3). Statistical differences were observed among F_2_ and F_3_ generations for non-recombined rDNA-bearing and non-rDNA-bearing chromosomes for both *F. pratensis* and *L. perenne*, as well as for recombined non-rDNA-bearing chromosomes of both genomes, but only for F_3_ generation. In this analysis, we also considered the comparison between analyzed generations. Statistically significant differences were identified for non-recombined rDNA-bearing chromosomes of both genomes and non-recombined non-rDNA-bearing chromosomes of *F. pratensis* genome, as well as for the number of recombined rDNA- and non-rDNA-bearing chromosomes for both genomes (Table 3). Additionally, the analysis of rearrangement locations revealed statistically significant differences in arms without rDNA markers (Table 3).

**Table 3:** Comparison of distributions of selected cytogenetic traits (recombined and non-recombined chromosomes belonging to *F. pratensis* and *L. perenne* genomes) within and between F_2_ and F_3_ generations of *F. pratensis* × *L. perenne* hybrid

Trait	Comparison F_2_ combination no. 1 vs. F_3_ generation	Comparison Lp vs. Fp in F_2_ generation	Comparison Lp vs. Fp in F_3_ generation
Lp chromosomes – non-recombined M+	**0.023**	**< 0.001**	**< 0.001**
Fp chromosomes – non-recombined M+	**< 0.001**		
Lp chromosomes – non-recombined M-	0.502	**< 0.001**	**0.020**
Fp chromosomes – non-recombined M-	**0.004**		
Lp chromosomes – recombined M+	**< 0.001**	1.000	0.201
Fp chromosomes – recombined M+	**0.002**		
Lp chromosomes – recombined M-	**0.020**	0.550	**< 0.001**
Fp chromosomes – recombined M-	**< 0.001**		
Lp chromosomes – arm recombined m+	0.133	0.185	**0.020**
Fp chromosomes – arm recombined m+	0.888		
Lp chromosomes – arm recombined m-	**< 0.001**	0.167	0.357
Fp chromosomes – arm recombined m-	**0.002**		

(Lp) *L. perenne*; (Fp) *F. pratensis*; (M+) rDNA-bearing chromosome; (M-) non-rDNA-bearing chromosome; (m+) chromosome arm with rDNA sequence; (m-) chromosome arm without rDNA sequence; bold *P*-values for comparisons considered as statistically significant

### ISSR loci polymorphism between parental plants

The ISSR analysis resulted in a reliable amplification of 71 loci in parental plants (*F. pratensis* and *L. perenne*). The total number of loci amplified in subsequent generations was lower due to the occurrence of recessive genotypes in both F_1_ and F_2_ parental individuals. Each ISSR primer produced markers with similar informativeness, as calculated by Polymorphism Information Content (PIC) for each of the parental, F_1_ and F_2_ individuals, as well as for each of F_2_ and F_3_ cross combinations (see Additional file 3: Table S2). The highest level of polymorphism, accounted for nearly 72% of loci, was observed between *F. pratensis* and *L. perenne* parental forms. The frequency of polymorphic loci in F_1_ generation was lower than in parental forms, although the individuals used for combination no. 2 were more polymorphic compared with the parental individuals of combination no. 1. Similarly, the polymorphism between individuals from F_2_ generation was further reduced, but F_2_ parents used for combination no. 4 showed more diversity than those used for combination no. 3 (Table 4). This finding was also reflected by the mean values of PIC, which showed that ISSR markers detected in the parents of cross combination 2 and 4 were more informative, and as a result higher mean value of PIC was also found for the progeny of these cross combinations (see Additional file 3: Table S2).

**Table 4:** ISSR polymorphism between parental species – *F. pratensis* and *L. perenne* and between pairs of the F_1_ and F_2_ plants used as parents for subsequent generations of *F. pratensis* × *L. perenne* hybrid

Compared genotypes	No. of monomorphic loci	No. of polymorphic loci	Polymorphism [%]
Fp 11/59 and Lp 08/33	20	51	71.83
F_1_ 6-2A and F_1_ 6-7A	51	15	22.72
F_1_ 6-2A and F_1_ 6-3B	37	30	44.77
F_2_ 2A-15 and F_2_ 2A-5	55	5	8.33
F_2_ 2A-27 and F_2_ 2A-12	51	8	13.55

### Segregation of ISSR loci in F_2_ and F_3_ generations

More than one type of the segregation ratio passed the χ^2^ test in the majority of ISSR loci (43 loci) in the combination no. 1 of F_2_ generation (see Additional file 4: Table S3). For 25 loci only one, non-distorted segregation ratio was noticed and in the case of two loci a segregation distortion was found. Additionally, one locus passed the χ^2^_13:15_ test, indicating its tetrasomic inheritance and the possibility of quadrivalents formation and random chromatid segregation. In the F_2_ combination no. 2, different proportion of each of those categories was recorded: for 33 loci only one non-distorted segregation type was found, in case of 27 loci more possibilities were detected and 11 ISSR loci showed distorted segregation (see Additional file 5: Table S4). Regardless of the above differences, similar proportions of particular inheritance types of ISSR loci were found in both F_2_ combinations. The tetrasomic inheritance was significantly prevailing over the disomic inheritance. Furthermore, the χ^2^ test results suggested that three loci (L7_300, L8_300, and L8_1400) have been inherited according to a different model in both F_2_ combinations (see Additional file 6: Table S5). Importantly, it was not possible to reliably assign the inheritance type to the majority of loci what may somehow bias these calculations (Table 5, see Additional file 6: Table S5). In these loci where distorted segregation was detected, the comparable proportion was skewed toward a dominant or recessive allele. In half of these cases, it was possible to point to the prevalence of the allele from specific parental species, and again similar proportion of distorted loci were skewed toward *Festuca* or *Lolium* allele, respectively (see Additional file 6: Table S5).

**Table 5:** Number of ISSR loci that showed particular inheritance type in F_2_ and F_3_ generations of *F. pratensis* × *L. perenne* hybrid

Cross combination	No. of loci		
	Disomic inheritance	Tetrasomic inheritance	Unknown
F_2_ combination no. 1	3	19	49
F_2_ combination no. 2	5	18	48
F_3_ combination no. 3	2	0	58
F_3_ combination no. 4	0	2	57

The similar analysis of F_3_ generation gave much less reliable results. This was caused by a significantly lower ISSR polymorphism between F_2_ plants taken as parents for the crosses. In the majority of loci, both lines were characterized by the presence of ISSR band making the genotype assignment more difficult. In addition, in 12 and 11 loci no bands amplification was observed in combination no. 3 and 4 of F_3_, respectively. The great majority of loci showed more than one possible segregation ratio in both F_3_ combinations (49 and 45 loci, for combinations no. 3 and 4, respectively). Three loci had distorted segregation in the combination no. 3 – one locus was skewed toward the recessive allele and two toward the dominant one. Five loci were distorted in the combination no. 4 – here four were skewed toward recessive allele and only one toward the dominant allele. In case of two loci, it was possible to point to the prevalence of the allele from specific parental species and in both of them, the distortion was toward *Lolium* allele (see Additional file 7: Table S6 and Addititional file 8: Table S7). When the occurrence of distorted loci was compared between F_2_ and F_3_ generation no specific pattern was noticed. Only one locus with distorted segregation in F_2_ generation was further distorted in the F_3_. In the remaining loci, no co-occurrence of such events in both generations was found. Moreover, the ISSR analysis of F_3_ generation did not allow to discover the inheritance type for the vast majority of loci (Table 5, see Additional file 9: Table S8).

**Fig. 5:**
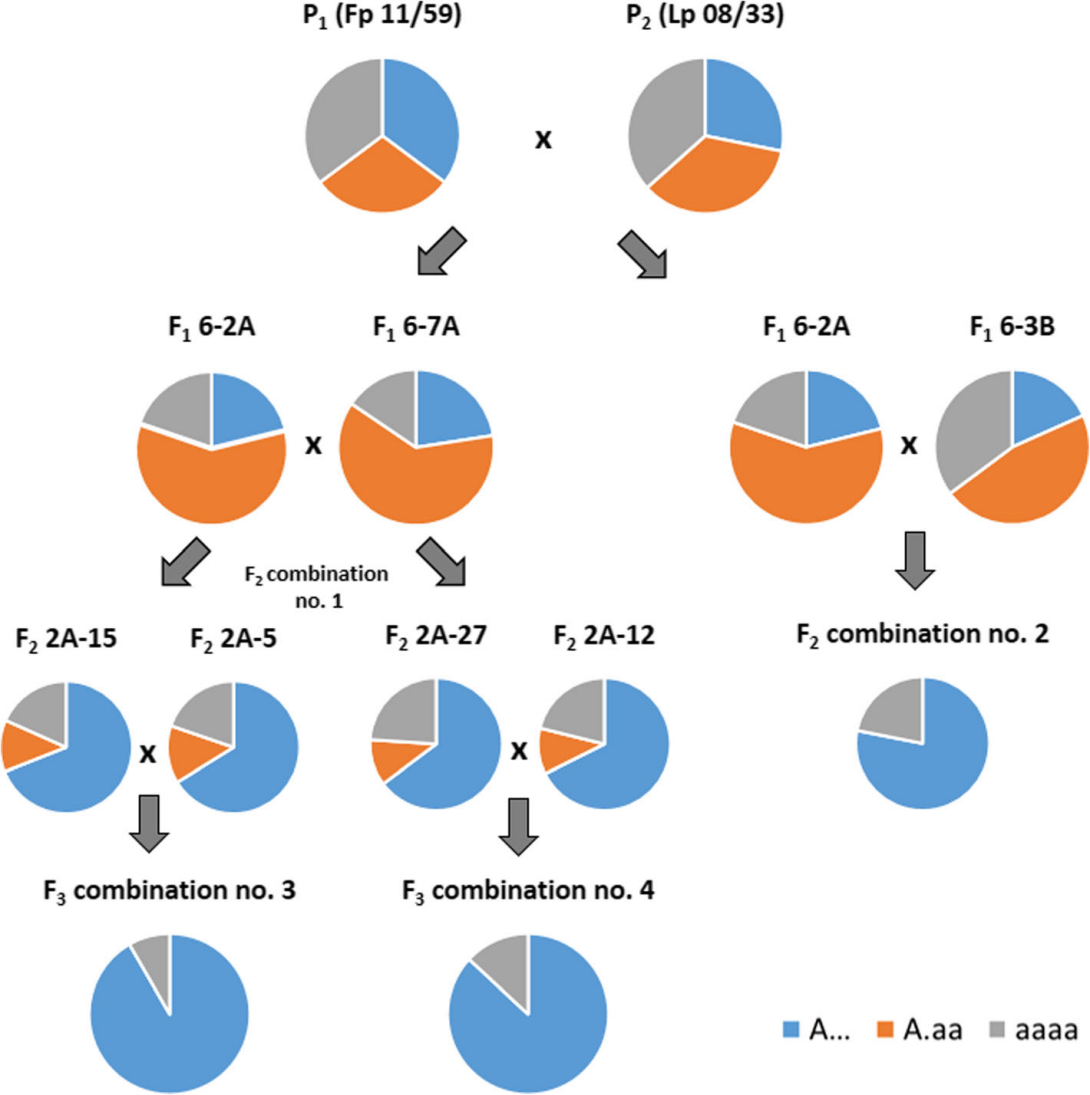
The distribution of the most probable genotypes that was inferred based on the segregation of ISSR loci for parental genotypes, F_1_ parents of both cross combinations and the F_2_ parents of the cross combination no. 1. In case of ISSR loci segregation in F_2_ generation from cross combination no. 2 and both F_3_ generations, only the frequency of precedence (dominant) or absence (recessive, nulliplex) of ISSR band was taken into account

In addition to the above analysis, the distribution of the frequencies of the most probable genotypes that was inferred based on ISSR loci segregation was provided (Fig. 5). It shows the gradual prevalence of dominant allele frequency in subsequent generations. Such observation may further support the tetrasomic type of ISSR inheritance, as in this type the dominant class is usually much numerous than the recessive class for the same cross combination of parental genotypes. It may also reflect the process of more frequent transmission of a dominant allele from generation to generation.

## Discussion

The formation of new synthetic allopolyploid plants via hybridization of different species can include: chromosomal rearrangements [17, 20], transposon activations [21–23], changes in rDNA loci [24, 25] and/or loss of DNA [26–28]. In allopolyploids, like allotetraploid *F. pratensis* × *L. perenne* hybrids, in which two genomes/species from different genera are merged, the deciphering of those processes is crucial to produce more persistent high-quality grass cultivars. For that purpose, the comprehensive analysis, including the examination of genome composition and structural reshufflings, the segregation of ISSR loci and their inheritance patterns in F_2_-F_3_ generations of allotetraploid *F. pratensis* × *L. perenne* hybrid was performed.

### Genomic structure of hybrids

In rDNA-FISH experiments, the same number and distribution of 5S rDNA were observed in the vast majority of studied F_2_ and F_3_ generations (in 131 out of 134 plants). Only in three plants, the lower or higher number of 5S rDNA loci was found. Nonetheless, this phenomenon was related to the changed total number of chromosomes in the complement. The more considerable variation was noticed for the number of 35S rDNA that ranged from seven to eleven loci in F_2_ and F_3_ plants, and the statistical analysis of this trait revealed significant differences between both generations, especially for the number of chromosomes 1, 2, 7L and 2F. It has been observed for many different plant species and hybrids that the number of 5S rDNA is stable, whereas 35S rDNA sequence seems to be more polymorphic [29, 30], what reflects showed rDNA patterns among studied *F. pratensis* × *L. perenne* hybrids. Nevertheless, Książczyk et al. [17] described the unstable number (from four to six hybridization signals) of 5S rDNA in F_2_-F_4_ generations of *F. pratensis* × *L. perenne* hybrid. In this plant material, the additional smaller hybridization sites were located in different regions of *F. pratensis* chromosomes and this was not related to the changed total number of chromosomes. Pedrosa-Harand et al. [31] suggested that variability in chromosomal positions, numbers and signal sizes of rDNA can be caused by at least three different mechanisms: (*i*) structural rearrangements that affect the position of rDNA loci; (*ii*) amplification of satellite DNA with no impact on the order of other sequences in chromosomes; and (*iii*) dispersion of rDNA repeats, amplification of the new minor loci and deletion of original major loci. The first two mechanisms are connected with changes in the chromosomal position of rDNA sequences, and the third one is responsible for changing the number and size of rDNA loci. Chromosomal re-patterning can be also connected with mobile elements activity [32], such as – *Enhancer/Suppressor-mutator* (*En*/*Spm*) transposons in *Aegilops* species [33] or *Wukong* terminal repeat retrotransposons in miniature (TRIM) in maize germplasm [34]. In interspecific hybrids, especially in newly synthesized hybrids, the targets of structural reconstructions can be rDNA sequences [35], what was also found in our plant material. In these plants, the rearrangements between *F. pratensis* and *L. perenne* genomes in/or near rDNA position took place. Huang et al. [36] reported that fragile sites (FSs) in chromosomes of *L. perenne* are linked with 35S rDNA cluster. The FSs are determined as regions that are prone to forming gaps or breaks on chromosomes. Human fragile sites are known as hotspots for chromosome aberrations, especially in many tumor cells [37, 38]. However, in *Festuca* × *Lolium* plants, the analysis of the relation between FSs associated with 35S rDNA regions and the location of structural rearrangements revealed that intergenomic changes did not correspond to FSs [39].

In theory, chromosomal rearrangements should not occur in allopolyploids due to preferential homologous chromosome pairing and sophisticated molecular processes preventing homoeologous recombination, such as *Ph1* locus in wheat [40] or pairing regulators in *Festuca*-*Lolium* complex [41–44]. It is worth to highlight the differences between pairing control genes in both genera – in *Festuca* species genes are haplo-insufficient, while in *Lolium* genus this genetic system is effective both at the hetero- and homozygous stage [45]. Nevertheless, closely related plant species usually display the synteny of genomes and the collinearity of gene order, what can contribute to mispairing [46]. In the analyzed F_2_-F_3_ generations of *F. pratensis* × *L. perenne*, the recombinations between both genomes took place. Rearrangements that were recognized in F_2_ progeny are in contrary to the idea of Ramsey and Schemske [47]. According to their theory, homoeologous pairing is suspected to be avoided during meiosis in the F_1_ generation following interspecific hybridization, because it can lead to infertility of progeny. Additionally, the increasing number of structural rearrangements in the next generations of *F. pratensis* × *L. perenne* hybrid was observed, what is consistent with our previous findings [17, 48, 49]. Beyond the frequent and increasing occurrence of homoeologous rearrangements among F_2_-F_3_ plants, we identified also the examples of non-homologous recombination. This type of recombination between non-rDNA bearing chromosome of *F. pratensis* and region of chromosome 3L (chromosomal segment bearing 5S rDNA locus) took place (Fig. 3). Non-homologous chromosome pairing and recombination could be provoked by cross hybridization and rapid genome changes. It is thought that both types of recombination probably play a significant role in genome reshuffling and stabilization. Genomes of many polyploids, such as *Triticum* [50], *Aegilops* [51], *Kengyilia* species [52] or × *Triticosecale* [53] underwent genome reshufflings during the course of evolution (especially translocation, deletions and inversions). Chromosomal aberrations are mainly located in heterochromatic regions composed of repetitive DNA. The analysis of rearrangement distribution in *F. pratensis* × *L. perenne* amphiploid forms revealed terminal location as the most frequent (see Additional file 2: Table S1). The recombination rates along individual *Festuca* and *Lolium* chromosomes evaluated by Kopecký et al. [54] and King et al. [55] revealed that recombination sites are concentrated in intercalary regions of chromosome arms and reduced in their proximal and distal parts.

rDNA-FISH combined with GISH approach allowed us to unequivocally discriminate selected rDNA-bearing chromosome pairs in the complement of analyzed plant material. For these pairs of chromosomes, the analysis was more detailed and revealed, which pair was the most recombined. Knowledge about the types of rearrangements and their frequencies is essential to better understand the evolutionary significance of genome reshufflings [56]. In both studied generations, the high frequency of recombination was calculated for *Festuca* rDNA-bearing chromosomes, in F_2_ plants – for chromosome 2F, while in F_3_ plants – for chromosome 3F. The high value of rearrangements in chromosome 2F was also depicted by Majka et al. [39] in F_2_-F_9_ generations of *F. pratensis* × *L. perenne* hybrid*.* Furthermore, the recognition of a particular chromosome pair revealed that in F_3_ prevailed plants with only one chromosome 2F. This odd number of the chromosome was inherited from parental forms – maternal plant had two chromosome 2F, while the pollinator did not have this pair of chromosomes. This unequivocal number of chromosomes 2F was mainly related to a proper (2n = 28) number of chromosomes. The loss of chromosome 2F could be also associated with deletion of 35S rDNA sequence or translocation of 35S rDNA into another chromosome. Jumping of rDNA sequences, especially 35S rDNA, is not the rare case in the plant kingdom [30, 33, 57].

The evolution of plant genomes, especially hybrids (including newly created by human and ancient natural hybrids) is related to genome and chromosome repatterning. In hybrid plants, the extensive interactions between genomes are observed. For hybrids derived from *Festuca*-*Lolium* complex (eight successive generations of *F. pratensis* × *L. perenne* hybrid), Zwierzykowski et al. [48, 49] observed preferential transmission of *Lolium* chromosomes, probably due to material selection for agronomic traits. However, the comparison of selected and unselected plants of F_2_-F_4_ generations of *F. pratensis* × *L. perenne* hybrid confirmed the dominance of *Lolium* over *Festuca* chromatin [58]. Our material was composed of plants without any selection and we also observed a higher number of *Lolium* chromosomes and drift in favor of *Lolium* in successive generations. Statistical analysis confirmed our hypothesis that asymmetrical variation between *F. pratensis* and *L. perenne* parental genomes exists. Nonetheless, Akiyama et al. [59] revealed no preferential transmission of the *Lolium* genome in consecutive generations of *L*. *muliflorum* (2x) × *F. arundinacea* (6x) hybrids.

### The inheritance of ISSR loci

During meiosis in autopolyploids, chromosomes may form multivalents leading to multisomic inheritance, where all the possible allelic combinations are formed in equal frequencies. In allopolyploids chromosomes usually pair with their homologs and form bivalents resulting in disomic inheritance. It has been observed, however, that both auto- and allopolyploids may shift between those two modes of inheritance and show intermediate or mixed inheritance [47], in which not all chromosomes have the same pairing behavior [6]. In addition, in the tetraploids two types of meiotic segregation may occur: random chromosome or random chromatid segregation, depending on the formation of bivalents or quadrivalents, respectively.

Our results suggest that in the F_1_ hybrids most of ISSR loci segregated to gametes in a tetrasomic way, although the relatively small number of F_2_ individuals did not allow us to determine which type of assortment: chromosome or chromatid might take place during meiosis of the F_1_ hybrid. There was just one locus, which segregation suggested the possibility of quadrivalents formation and random chromatid segregation. Only in a few loci, disomic segregation could be accepted based on the χ^2^ test result. Their occurrence, however, proved the possibility of a mixed model of inheritance in *Festulolium* hybrid plants. From ISSR data only, it is not possible to decide whether there is any specific preference of chromosome pairing in particular chromosome homologue or homoeologue group. Nevertheless, from the nature of ISSR marker system, it may be assumed that the ISSR loci represent random positions in the genome and will be located in different chromosomes, suggesting that most of the chromosomes in F_1_ hybrids may form intergenomic bivalents or multivalents during meiosis. As indicated by Zwierzykowski et al. [60] who studied the meiosis of *F. pratensis* × *L. perenne* hybrids, the *F. pratensis*/*L. perenne* intergenomic bivalents were observed in about 33% of analyzed cells. A significant number of quadrivalents and some trivalents or univalent were also found. Such observations support the possibility of tetrasomic way of inheritance of ISSR loci, although, further analysis, for example with the use of ISSR amplification products as probes for in situ hybridization, may give more inside into this problem and shed more light on the chromosome vs. chromatid model of assortment of genetic material into the gametes.

An interesting observation was the occurrence of three ISSR loci, which seem to be inherited in different ways in both F_2_ combinations. It may suggest that the choice between bivalent or multivalent pairing, at least for some chromosomes in the F_1_ meiosis may be equally possible and take place at random.

Multivalent formation followed by a crossing-over located between the centromere and particular locus may in some cases lead to the migration of sister alleles to the same gamete during meiosis – a phenomenon known as a double reduction [61]. It may have a role in the purging of deleterious mutations through gametophytic selection [62], and could affect the distribution of gene frequency in polyploid populations [63]. In our study, a decline of ISSR polymorphism was found between subsequent generations. We understand that the dominant nature of ISSR markers does not allow to precisely measure the diversity, especially in tetraploid species, but it cannot be excluded that double reduction together with other mechanisms may be one of the reasons for this observation.

Our analysis of F_3_ plants shows that the mixed model of the inheritance probably persists in the subsequent generation, although this conclusion may only be speculative as almost all ISSR loci in F_3_ generation were not assigned to any specific model of inheritance. This high proportion of ISSR loci with an unknown type of inheritance, is another important factor to consider, as it may in some way bias our analysis. We cannot exclude, however, that irregular cytogenetic behaviors, such as the formation of univalents and trivalents, as evidenced in Zwierzykowski et al. [60] may contribute to the events with unknown inheritance mode. Here, further extending the sample size or the application of co-dominant markers and ISSR-FISH analysis may allow to specify the type of inheritance in *F. pratensis* × *L. perenne* hybrids.

### Segregation distortion of ISSR loci

Our ISSR analysis showed that only the minority of ISSR loci in F_2_ and F_3_ generations were characterized by segregation distortion (SD). They accounted for about 3.0% and 15.0% in two F_2_ cross combinations and for 5.0% and 8.4% in the two F_3_ cross combinations. We are aware, however, that we might underestimate the frequency of this phenomenon, as the dominant type of ISSR markers does not allow to fully assign the genotypes at particular loci and, consequently, to test only these segregation ratios that are unambiguous. The possibility of underestimation of this result may be suggested by other studies of *Festuca* and/or *Lolium* hybrids. For example, the genetic mapping studies in *L. perenne* showed that depending on the mapping population used, the proportion of distorted loci ranged from 12% [64] to 60% [65]. In the mapping study of *F. arundinacea*, segregation distortion was observed from 23% [66] to 45% of loci [67]. The mechanisms underlying SD are only partially understood and may involve various processes, such as differential inclusion of parental alleles into gametes (meiotic drive), differential fertilization success of specific gametes or differences in survival of haploid gametes or zygotes with specific allelic composition [68]. Some evidence suggests that SD is higher in interspecific than in intraspecific crosses [69, 70] thus being a reflection of more dynamic changes in the interspecific hybrids genomes.

The use of ISSR markers in our study suggest that there were no preferences in the transmission of either *Festuca* or *Lolium* ISSR alleles to the following generations of *F. pratensis* × *L. perenne* hybrid, nor there were any preferences either for transmission of the dominant or recessive allele. Some preferences toward the transmission of the recessive allele were noticed in the F_3_ combination no. 4, although it may be related to the relatively low number of loci under SD detected, and happened by a chance only. However, one concurrence may be noticed – more distorted loci were found in these populations, which parents were more polymorphic. Successful amplification of ISSR locus relies on two factors: (*i*) the presence of two microsatellite sequences in the reverse complement orientation in DNA strand that are complementary to the oligonucleotide used for the PCR reaction, and (*ii*) the location of such sequences in a distance short enough to be amplified by routinely used polymerases and PCR assays. If the second mechanism will be considered, the lack of ISSR band will be a result of the too long distance between priming sites. This opens a possibility to think about the relationship between the length of *Festuca* and *Lolium* chromosomes and their possibility of successful pairing during meiosis. Distorted segregation may occur in these loci where an imbalance of a homoeologous chromosome segment length is present. Such hypothesis assumes that these chromosomes pair as multivalents and further suggests the intermediate or mixed model of inheritance in *Festuca* × *Lolium* hybrids.

## Conclusions

The cytogenetic analysis of 134 plants of F_2_-F_3_ generations of *F. pratensis* × *L. perenne* hybrid, including rDNA-FISH and GISH together with molecular genotyping of all plants by ISSR markers allowed us to draw conclusions from data reported herein. Cytogenetic genotyping of all analyzed hybrid plants revealed discrepancies in parental genomes (*Festuca* and *Lolium*) behavior. Statistical analysis of that data implies a genome drift over successive generations in allotetraploid *F. pratensis* × *L. perenne* hybrid. We observed the increasing number of *Lolium* chromosomes in the next generations of hybrids and significant differences between F_2_-F_3_ generations for recombined rDNA-bearing *L. perenne* chromosomes. Despite the decreasing total number of *F. pratensis* chromosomes, their chromosomes were more rearranged and statistical differences between analyzed generations for recombined rDNA-bearing and non-rDNA-bearing ones were recognized. Nevertheless, genotyping by ISSR markers analysis evidenced no preference in the transmission of either *Festuca* or *Lolium* alleles to the following generations of *F. pratensis* × *L. perenne* hybrid. It is important, however, to bear in mind the different resolutions of both cytogenetic and molecular markers analysis. Furthermore, the analysis of ISSR loci inheritance in these hybrid plants revealed that the tetrasomic type of inheritance was characteristic for the majority of ISSR loci, but in some cases, the disomic type was also observed. This indicates that the mixed or intermediate model of alleles transmission may occur in the hybrids. Such possibility is in the agreement with the detection of non-homologous recombination events, as the multivalent formation may promote rearrangements between chromosomes from different genomes. Further examinations of the possible rearrangements combining chromosome and DNA level of resolution may give deeper insight into the nature of how the genome balance is achieved in *F. pratensis* × *L. perenne* hybrid.

## Methods

### Plant material

Allotetraploid F_1_ plants (2n = 4x = 28) were produced by crossing the autotetraploid (2n = 4x = 28) *F. pratensis* Huds. (cv. ‘Westa’; Fp 11/59) (♀) with the autotetraploid (2n = 4x = 28) *L. perenne* L. (cv.‘Solen’; Lp 08/33) (♂). The F_1_ maternal plant (F_1_ 6-2A) and two different F_1_ pollinators were used to produce two combinations of the F_2_ generation: no. 1 – with F_1_ pollinator 6-7A and no. 2 – with F_1_ pollinator 6-3B. The F_3_ generation (combinations no. 3 and 4) was obtained by crossing four randomly selected F_2_ plants derived from combination no. 1. The detailed scheme of crossing experiments was presented in Additional file 1: Fig. S1. All plant material derived from the collection of the Institute of Plant Genetics, Polish Academy of Sciences in Poznan, Poland.

### Preparation of root meristems

Roots from plants growing in a greenhouse were excised and pretreated in ice-cold water for 24 h, fixed in 3:1 (v/v) ethanol:glacial acetic acid and then stored at − 20 °C until required. Mitotic chromosome preparations were made using the excised roots, according to the procedure described in Majka et al. [71]. The high-quality slides were frozen and stored at 4 °C until used.

### Fluorescent in situ hybridization

The FISH procedure was carried out according to the protocol published in Majka et al. [71]. The hybridization mixture consisted of 50% deionized formamide, 10% dextran sulfate, 2 × SSC, 0.5% SDS, 10 μg salmon sperm blocking DNA and 100-120 ng/slide both rDNA probes. In FISH experiments the following probes were used: (*i*) the 5S rDNA sequence, generated from the wheat clone pTa794 [72] and (*ii*) 35S rDNA sequence, generated from a 2.3-kb *Cla*I sub-clone of the 25S rDNA coding region of *Arabidopsis thaliana* [73]. 5S rDNA was labeled by PCR technique with universal M13 primers by using tetramethyl-rhodamine-5-dUTP (Roche, Mannheim, Germany), while 35S rDNA probe was prepared by nick translation with digoxigenin-11-dUTP (Roche, Mannheim, Germany). Chromosome preparations were denatured at 80 °C for 2 min in presence of hybridization mixture and hybridized overnight in a humid chamber at 37 °C. Post-hybridization washes in 0.1 × SSC at 42 °C provided an equivalent of 79% stringency. Digoxigenin-labeled probes were detected by fluorescein isothiocyanate-conjugated (FITC) anti-digoxigenin antibody (Roche, Mannheim, Germany). Chromosomes were counterstained with 2.5 mg/ml 4′,6-diamidino-2-phenylindole (DAPI, Sigma, St. Louis, Missouri) in antifade Vectashield solution (Vector Laboratories, Burlingame, CA, USA).

The chromosome nomenclature used for rDNA-bearing chromosomes was developed by Thomas et al. [74]. According to this, there are two rDNA-bearing chromosomes in *F. pratensis* karyotype: chromosomes 2F (bearing 35S rDNA loci) and 3F (bearing 5S rDNA). Whereas in the *L. perenne* karyotype, chromosomes 3L (bearing 5S and 35S rDNA in opposite arms) and a group of chromosomes 1, 2 and 7L (bearing 35S rDNA) can be recognized.

### Genomic in situ hybridization

For the GISH experiments all the chromosome spreads, which had been used in FISH, were applied. Therefore, before GISH, the sets of probes from previous experiments were rinsed off. The GISH procedure was carried out according to the protocol of Majka et al. [39]. Genomic DNA of *L. perenne*, which was mechanically sheared to fragments of 5-10 kb by boiling for 30-45 min and labeled by nick translation with digoxigenin-11-dUTP (Roche, Mannheim, Germany), was used as a probe in GISH experiments. While the nuclear DNA of *F. pratensis* was used as a blocking DNA (sheared to the shorter fragments by boiling for 10-15 min). The probe was detected using a FITC conjugated anti-digoxigenin antibody. The chromosome preparations were counterstained in Vectashield (Vector Laboratories, Burlingame, CA, USA) containing 1.5 g/ml^− 1^ of propidium iodide.

### Image capturing and data processing

Images were captured using an Olympus XM10 CCD camera attached to an Olympus BX 61 automatic epifluorescence microscope. Final image adjustments were done with Olympus Cell-F imaging software (ver. 3.1; Olympus Soft Imaging Solutions GmbH, Germany) and Micrographx Picture Publisher software (ver. 10; Corel Corporation, Canada).

Cytogenetic data were statistically processed by the χ^2^ test and t-test within GenStat® (ver. 16; VSN Int.). Analysis was performed to compare the distributions of selected cytogenetic traits within and between F_2_ and F_3_ generations, as well as to evaluate the difference between both parental genomes in F_2_-F_3_ generations of *F. pratensis* × *L. perenne* hybrid. The comparison between F_2_ and F_3_ generations was performed for the F_2_ cross combination no. 1 only, because of crossing scheme – combinations no. 3 and 4 (F_3_ generation) were obtained by crossing plants that derived from combination no. 1 of F_2_ generation.

### DNA isolation and ISSR-PCR analysis

DNA samples were extracted from young leaves using CTAB method [75]. ISSR-PCRs were carried out in a volume of 25 μl containing 70 ng of genomic DNA, 2 × PCR buffer, 2.5 mM MgCl_2_, 2.5 mM dNTP, 10 mM of each primer and 0.25 units of Taq DNA Polymerase (Thermo Scientific, Waltham, Massachusetts). The thermal profile for ISSR-PCR was as follows: 94 °C for 2 min – initial denaturation, then 35 cycles of 95 °C for 30 s, the temperature of annealing was depended on primer (48–57 °C) for 1 min and elongation at 72 °C for 1 min. A final extension step – 5 min at 72 °C. The temperature of annealing and sequences of analyzed oligonucleotides were shown in Additional file 10: Table S9. ISSR amplification products were separated in 2.0% agarose gels (EURx, Gdańsk, Poland), stained with ethidium bromide (Sigma, St. Louis, Missouri) and visualized under UV light and photographed (Syngen UV visualizer). GeneRuler Ladder (Novazym, Poznań, Poland) was used to determine the size of the DNA fragments.

### The analysis of ISSR loci polymorphism

In order to estimate the level of polymorphism between parental plants: *F. pratensis* (Fp 11/59) and *L. perenne* (Lp 08/33) and between pairs of the F_1_ and F_2_ individuals used as parents for subsequent generations, all the bands produced by each ISSR primer were counted. The entire set of bands was composed of monomorphic products present in both analyzed forms and polymorphic products characteristic for individual genotypes. The level of polymorphism was defined as the percentage of polymorphic bands for all the primer combinations (see Additional file 11: Table S10 and Additional file 12: Table S11). The Polymorphism Information Content (PIC) was calculated using iMEC program [76].

### The analysis of ISSR loci segregation

Based on the presence or absence of ISSR band, the possible genotypes were assigned to parental *F. pratensis* and *L. perenne* plants and to the F_1_ and F_2_ genotypes which were used as parents for subsequent cross combinations. As both parental plants (Fp 11/59 and Lp 08/33) are autotetraploids, we assumed that their chromosomes will segregate to gametes in a tetrasomic way and we have taken into account the possibility of random chromosome or random chromatic segregation. The individuals of F_1_ and F_2_ generation were allotetraploids, but our observations suggest that both tetrasomic and disomic inheritance of chromosomes is possible, although it was found that bivalent formation is more frequent in the hybrid [77]. Taking into account this observation as well as the dominant type of ISSR markers that were used in our study and the relatively low sample size, which does not allow to distinguish between chromosome vs. chromatid assortment, we made most of the later calculations based on the simplified assumption of the random chromosome segregation model. This allowed us to divide the types of inheritance into tetrasomic and disomic, based on the different possible rates of band segregation, depending on the genotypes in each ISSR locus in parental individuals (see Additional file 13: Table S12). All possible segregation ratios for random chromosome segregation model were tested using the χ^2^ test. We assumed that the lack of segregation distortion is more probable than its occurrence. In all cases, where only one segregation ratio passed the χ^2^ test (*P* < 0.05), the one was selected as describing the most probable inheritance type in particular ISSR locus. When more segregation rates passed the χ^2^ tests and some of them were characteristic to different inheritance types, we assumed such result as ambiguous with unknown inheritance type. When none of the segregation rates passed the χ^2^ test, such locus was treated as segregation distortion and a possible signature of genomic changes between parental plants and the subsequent F_2_ and/or F_3_ generations. To confirm the probability of segregation distortion of such locus it was additionally analyzed for those segregation ratios that are characteristic for random chromatid segregation model, which assumes quadrivalent formation (see Additional file 13: Table S12).

## Additional files

Additional file 1: **Figure S1.** The scheme of crossing experiments. (TIF 1581 kb)

Additional file 2: **Table S1.** Cytogenetic genotyping in *F. pratensis*, *L. perenne*, *F. pratensis* × *L. perenne* hybrids (F_1_) and in the following F_2_-F_3_ generations. (XLSX 85 kb)

Additional file 3: **Table S2.** The Polymorphism Information Content (PIC) calculated for individual ISSR primer and the PIC mean value. PIC values are provided for individuals that were used as parents for each of the cross combinations, as well as for the F_2_ and F_3_ populations. (XLSX 11 kb)

Additional file 4: **Table S3.** The results of χ^2^ test of ISSR bands segregation in combination no. 1 of F_2_ generation of *F. pratensis* × *L. perenne* hybrid. (XLSX 38 kb)

Additional file 5: **Table S4.** The results of χ^2^ test of ISSR bands segregation in combination no. 2 of F_2_ generation of *F. pratensis* × *L. perenne* hybrid. (XLSX 39 kb)

Additional file 6: **Table S5.** List of the most probable genotypes of P_1_, P_2_ and F_1_ parental plants for each of SSR locus. (XLSX 17 kb)

Additional file 7: **Table S6.** The results of χ^2^ test of ISSR bands segregation in combination no. 3 of F_3_ generation of *F. pratensis* × *L. perenne* hybrid. (XLSX 45 kb)

Additional file 8: **Table S7.** The results of χ^2^ test of ISSR bands segregation in combination no. 4 of F_3_ generation of *F. pratensis* × *L. perenne* hybrid. (XLSX 47 kb)

Additional file 9: **Table S8.** List of the most probable genotypes of P_1_, P_2_, F_1_, and F_2_ parental plants for each of ISSR locus. (XLSX 18 kb)

Additional file 10: **Table S9.** Specification of analyzed oligonucleotides. (XLSX 10 kb)

Additional file 11: **Table S10.** ISSR band pattern in combinations no. 1 and 2 of F_2_ generation of *F. pratensis* × *L. perenne* hybrid. (XLSX 40 kb)

Additional file 12: **Table S11.** ISSR band pattern in combinations no. 3 and 4 of F_3_ generation of *F. pratensis* × *L. perenne* hybrid. (XLSX 30 kb)

Additional file 13: **Table S12.** Possible segregation ratios for the disomic and tetrasomic inheritance of ISSR loci depending on the possible genotypes and band pattern in parental species – *F. pratensis* and *L. perenne* and in the F_2_-F_3_ generations of *F. pratensis* × *L. perenne* hybrid. For simplicity, the F_1_ genotypes were described using one letter code (A or a), regardless of the origin of particular allele from *Festuca* or *Lolium* parent. (XLSX 10 kb)

## Abbreviations

DAPI: 4′,6-diamidino-2-phenylindole
FISH: Fluorescent in situ hybridization
FSs: Fragile Sites
GISH: Genomic in situ hybridization
ISSR: Inter-Simple Sequence Repeats
PIC: Polymorphism Information Content
SD: Segregation Distortion
