# Supplementary figures and images for: Cytogenetic and molecular genotyping in the allotetraploid Festuca pratensis × Lolium perenne hybrids
Source: BMC Genomics. 2019 May 14;20:367. doi: 10.1186/s12864-019-5766-2 (PMC6518686; doi:10.1186/s12864-019-5766-2)

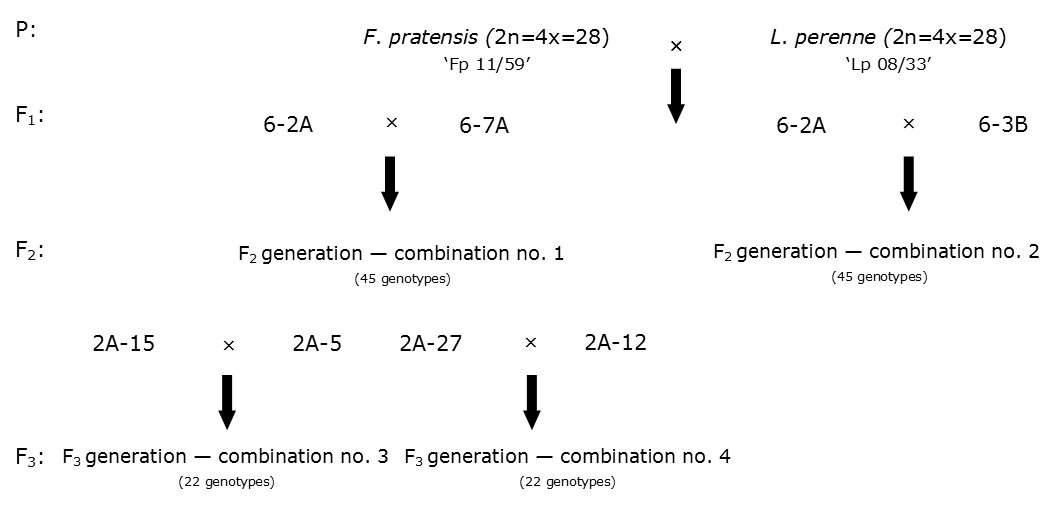

Supplement: Supplementary file 1 — Figure S1. The scheme of crossing experiments. (TIF 1581 kb) [file 12864_2019_5766_MOESM1_ESM.tif]
